# Supplementary material for: Neutralizing human monoclonal antibodies that target the PcrV component of the type III secretion system of Pseudomonas aeruginosa act through distinct mechanisms
Source: eLife. 2026 Feb 17;14:RP105195. doi: 10.7554/eLife.105195 (PMC12912723; doi:10.7554/eLife.105195)
Supplement: Supplementary file 1. — Percentage (%) of identity was obtained by aligning variable region sequences on IMGT database (https://www.imgt.org/). [file elife-105195-supp1.docx]

| **Heavy chain** | | **V Gene and allele** | **J Gene and allele** | **Anti-PcrV mAbs**  **V region identity (%)** | **J region identity (%)** | **CDR3 length** | **Donor #** |  |
| --- | --- | --- | --- | --- | --- | --- | --- | --- |
| **P3G9Hc P3D6Hc**  **P5B3Hc**  **P5E10Hc**  **Light chain** | | **IGHV4-61*08**  **IGHV3-23*01**  **IGHV3-74*01**  **IGHV3-21*01**  **V Gene and allele** | **IGHJ6*02**  **IGHJ4*02**  **IGHJ3*02**  **IGHJ6*02**  **J Gene and allele** | **92.44**  **92.70**  **96.88**  **88.42**  **V region identity (%)** | **91.94**  **89.58**  **94.00**  **74.20**  **J region identity (%)** | **18**  **14**  **11**  **12**  **CDR3 length** | **16**  **25**  **25**  **25**  **Donor #** |  |
| **P3G9κc**  **P3D6κc P5B3λc**  **P5E10κc**  **Heavy chain** | | **IGKV3-20*01**  **IGKV1-8*01**  **IGLV3-25*03 IGKV4-1*01**  **V Gene and allele** | **IGKJ3*01**  **IGKJ4*01**  **IGLJ2*01**  **IGKJ4*01**  **J Gene and allele** | **94.68**  **96.41**  **96.10**  **95.00**  **Anti-PscF mAbs**  **V region identity (%)** | **100.00**  **97.37**  **100.00**  **100.00**  **J region identity (%)** | **10 9**  **11**  **9**  **CDR3 length** | **16**  **25**  **25**  **25**  **Donor #** |  |
| **P1B7Hc**  **P1D5Hc**  **P1B4Hc**  **P1F5Hc**  **P1D7Hc P3G2Hc**  **P3G6Hc**  **P3G7Hc**  **P5D5Hc**  **P5G10Hc**  **Light chain** | | **IGHV3-23*01**  **IGHV4-61*02**  **IGHV3-23*01**  **IGHV3-23*01**  **IGHV3-23*01**  **IGHV4-61*02**  **IGHV3-23*01**  **IGHV3-72*01**  **IGHV3-23*01**  **IGHV4-34*01**  **V Gene and allele** | **IGHJ4*02**  **IGHJ6*02**  **IGHJ6*02**  **IGHJ6*02**  **IGHJ4*02**  **IGHJ4*02**  **IGHJ6*02**  **IGHJ3*01**  **IGHJ4*02**  **IGHJ6*02**  **J Gene and allele** | **97.22**  **83.85**  **95.14**  **77.86**  **96.87**  **91.40**  **98.61**  **86.39**  **96.52**  **91.22**  **V region identity (%)** | **91.67**  **80.65**  **83.87**  **83.87**  **93.75**  **93.75**  **85.48**  **88.00**  **93.75**  **77.42**  **J region identity (%)** | **13**  **19**  **17**  **19**  **15**  **16**  **17**  **18**  **13**  **16**  **CDR3 length** | **25**  **25**  **25**  **25**  **25**  **25**  **25**  **25**  **25**  **25**  **Donor #** |  |
|  | \| **P1B7κc**  **P1D5κc**  **P1B4κc**  **P1F5κc**  **P1D7λc P3G2λc**  **P3G6κc**  **P3G7λc**  **P5D5κc**  **P5G10λc** \| **IGKV1-16*02**  **IGKV2-28*01**  **IGKV3-20*01**  **IGKV2-28*01**  **IGLV3-21*02**  **IGLV2-8*01**  **IGKV3-20*01**  **IGLV1-51*01**  **IGKV1-16*01 IGLV1-47*01** \| **IGKJ2*01**  **IGKJ3*01**  **IGKJ1*01**  **IGKJ2*01**  **IGLJ2*01**  **IGLJ2*01**  **IGKJ2*01**  **IGLJ2*01**  **IGKJ2*01 IGLJ2*01** \| **97.13**  **94.22**  **95.04**  **95.58**  **98.56**  **94.79**  **99.60**  **88.00**  **98.20**  **94.73** \| **97.14**  **100.00**  **97.14**  **89.47**  **97.37**  **88.57**  **89.74**  **91.67**  **94.29**  **91.67** \| **9**  **9**  **9**  **17**  **11**   1. **9** 2. **9**   **11** \| **25**  **25**  **25**  **25**  **25**  **25**  **25**  **25**  **25**  **25** \| \| --- \| --- \| --- \| --- \| --- \| --- \| --- \| | | | | | | | |
