# Supplementary figures and images for: Neutralizing human monoclonal antibodies that target the PcrV component of the type III secretion system of Pseudomonas aeruginosa act through distinct mechanisms
Source: eLife. 2026 Feb 17;14:RP105195. doi: 10.7554/eLife.105195 (PMC12912723; doi:10.7554/eLife.105195)

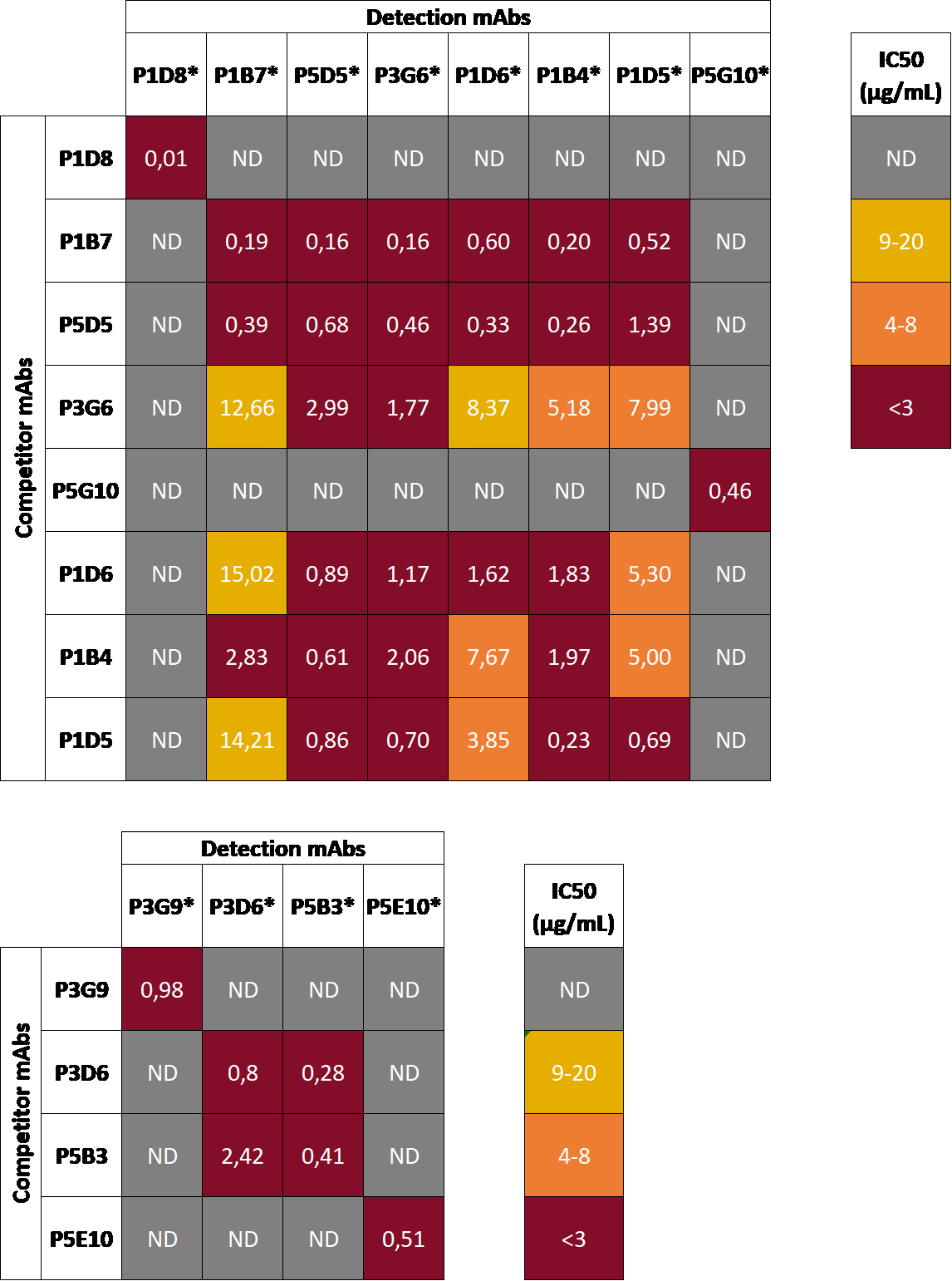

Supplement: Supplementary file 2. — The indicated IC50 values correspond to the concentration of competitor mAbs necessary to obtain half of the signal generated by the biotinylated mAbs without competitor. ND corresponds to a non-detectable competition. Source Data: Source data 2. [file elife-105195-supp2.png]

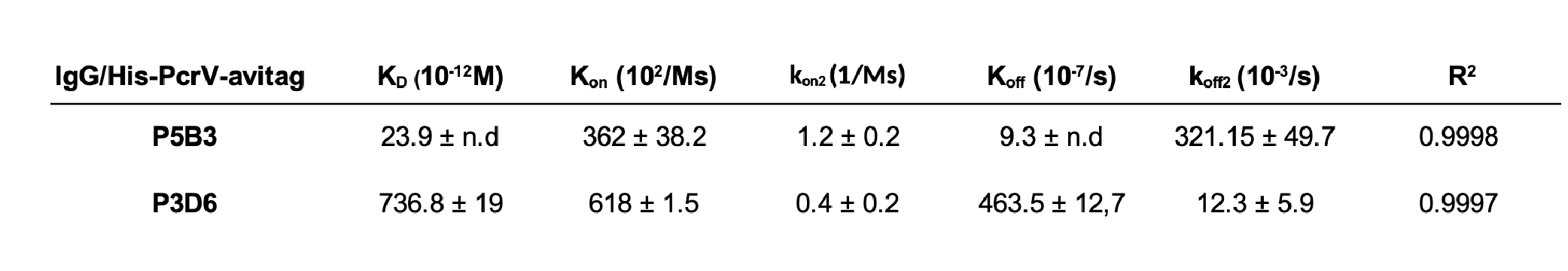

Supplement: Supplementary file 3. — The reported values correspond to the average of the measurements obtained from two independent experiments (n=2). Standard Deviations were calculated by the BLI analysis software. Source Data: Source data 2. [file elife-105195-supp3.png]
