# Supplementary material for: Neutralizing human monoclonal antibodies that target the PcrV component of the type III secretion system of Pseudomonas aeruginosa act through distinct mechanisms
Source: eLife. 2026 Feb 17;14:RP105195. doi: 10.7554/eLife.105195 (PMC12912723; doi:10.7554/eLife.105195)
Supplement: Supplementary file 4. [file elife-105195-supp4.docx]

**DATA COLLECTION**

| X-ray source (Beamline) |  | ESRF (ID30A-1) |
| --- | --- | --- |
| Detector |  | Pilatus 2M |
| Wavelength (Å) |  | 0.965459 |
| Scan-range (^o^) |  | 180 |
| Oscillation (^o^) |  | 0.2 |
| Space group |  | P2_1_ |
| ***a*** (Å) |  | 87.58 |
| ***b*** (Å) |  | 87.58 |
| ***c*** (Å) |  | 105.22 |
| a (^o^) |  | 90.00 |
| b (^o^) |  | 104.83 |
| g (^o^) |  | 90.00 |
| Mosaicity (^o^) |  | 0.129 |
| Overall resolution (Å) |  | 49.21-2.56 |
| No. observed/unique reflections |  | 176390/52157 |
| High resolution shell (Å) |  | 2.71-2.56 |
| Completeness (%) (last shell) |  | 99.5(99.2) |
| R*_sym_* (%) (last shell) |  | 21.7 (152.9) |
| *I/σ(I)* (last shell) |  | 5.02 (0.72) |
| CC1/2 |  | 97.7 (31.2) |
| Wilson plot B-factor (Å^2^)    **MOLECULAR REPLACEMENT** |  | 45.86 |
| Phaser LLG |  | 1032.93 |
| R*work*/R*free* (%)    **REFINEMENT** |  | 35.68/40.33 |
| Initial R*_work_*/R*_free_* (%) |  | 35.68/40.33 |
| Final R*_work_*/R*_free_* (%) |  | 19.69/23.64 |
| Final+TLS R*_work_*/R*_free_* (%)    **STEREOCHEMICAL QUALITY** |  | 20.97/23.70 |
| RMS deviation, bond lengths (Å) |  | 0.006 |
| RMS deviation, bond angles (º) |  | 1.266 |
| Mean B-factor (Å^2^) |  | 46.74 |
| No. of atoms |  | 9052 |
| No. of protein atoms |  | 8614 |
| No. of water molecules  Residues in most favored/allowed |  | 392 |
| region of Ramachandran plot (%) |  | 99.80 |
