# Supplementary material for: Neutralizing human monoclonal antibodies that target the PcrV component of the type III secretion system of Pseudomonas aeruginosa act through distinct mechanisms
Source: eLife. 2026 Feb 17;14:RP105195. doi: 10.7554/eLife.105195 (PMC12912723; doi:10.7554/eLife.105195)
Supplement: Supplementary file 5. [file elife-105195-supp5.docx]

| ***P. aeruginosa* strains** | **Characteristics** | **Reference or source** |
| --- | --- | --- |
| CHAΔ*exoS*::*exoS*-*bla* | β-lactamase (Bla) reporter for ExoS translocation | Verove et al., 2012 |
| CHAΔ*pcrV* | Strain lacking *pcrV* | Gouré et al., 2004 |
| PAO1Δ3Tox | Strain lacking toxins ExoS, ExoT and ExoY | Cisz et al., 2008 |
| **Plasmids** | **Characteristics** | **Reference or source** |
| pIApG-*pcrV*-V1 (PAO1) | Replicative plasmid for PcrV expression, *Leu^6^Ala^9^Ser^21^Ser^225^ | Lab collection; Simona Barzu, Lyon |
| pIApG-*pcrV*-V2 (CHA) | Leu^6^Ala^9^Ser^21^Arg^225^ | Lab collection; Simona Barzu, Lyon |
| pIApG-*pcrV*-V3 (PA14) | Phe^6^Ala^9^Pro^21^Lys^225^ | Lab collection; Simona Barzu, Lyon |
| pIApG-*pcrV*-V4 (PA103) | Phe^6^Gly^9^Pro^21^Arg^225^ | Lab collection; Simona Barzu, Lyon |
| pIApG-*pcrV*-V5 | Phe^6^Gly^9^Pro^21^Lys^225^ | Lab collection; Simona Barzu, Lyon |
| pET15b-His-PcrV | Replicative plasmid for inducible His-PcrV expression | Nanao et al., 2003 |
| pET22b-PscF-His | Replicative plasmid for inducible PscF-His expression | Quinaud et al, 2005 |
| pESPRIT-His-PcrV-avitag | Replicative plasmid for inducible His-PcrV-avitag expression  expression | This study |
| pESPRIT-His-PscF-avitag | Replicative plasmid for inducible expression His-PscF-avitag | This study |

*variable residues within PcrV
