# Supplementary material for: Neutralizing human monoclonal antibodies that target the PcrV component of the type III secretion system of Pseudomonas aeruginosa act through distinct mechanisms
Source: eLife. 2026 Feb 17;14:RP105195. doi: 10.7554/eLife.105195 (PMC12912723; doi:10.7554/eLife.105195)
Supplement: Supplementary file 6. [file elife-105195-supp6.docx]

| **Anti-PcrV mAbs** |  | **Variable domain nucleotide sequence** |
| --- | --- | --- |
| **P3G9** | Heavy chain | caggtgcagctgcaggaatcgggcccaggactggtgaagccttcggagactctgtccctcacctgcactgtctctaatgattccttcagcagtaatgattactactggaactggatccggcagcccccagggaagggactggagtggattggatatatctattacagtggtagcaccaactacaacccctccctcaagagtcgagtcaccatgtcggtagacacgtccatggatcagttctccctgaggctgagctctgtgacggctgcggacacggccgtttattactgcgcgagagggcgttacttcggtatctatcccaactactactacgctatggacgtctggggccaagggaccacggtcaccgtctcctcag |
|  | Kappa light chain | gaaattgtgttgacgcagtctccaggcaccctgtctttgtctccaggggaaaaagccaccctctcctgcagggccagtcagagtgtttccagcaactacttagcctggtaccagcagaaacctggccaggctccccggctcctcgtctatggttcatccagcagggccacaggcatcccagacaggttcagtggcagtgggtctggggcagacttcactcttcaaatcagcaaactggagcctgaagattttgcagtgtattactgtcagcagtacggtagctcgcccttattcactttcggccctgggaccaaagtggatatcaaac |
| **P3D6** | Heavy chain | caggtgcagctgttggggtctgggggaggcttggttcagccgggggggtcactgagactctcctgtgcagcctctggattcacctttcgcaactatgccatgatctgggtccgccaggctccagggaaggggctggactgggtctcaactattagtggtactggtgatagttcagactacgcagactccgtgaggggccggttcaccgtctccagagacaattccaagaacacgctgtatctggaaatgaacagcctgagagccgaagacacggccgttttttactgtgcgaaagattcgcggggggcagcagctgaattctttgactactggggccagggaatcttcgtcaccgtctcctcag |
|  | Lambda light chain | gccatccggatgacccagtctccatcctcattctctgcatctataggagacagagtcaccatcacttgtcgggcgagtcagggtattagcagttatttagcctggtatcagcaaaaaccggggaaagcccctaacctcctgatctatgctacatcctctttgcaaagtggggtcccatcaaggttcagcggcagtggatctgggacagatttcactctcaccatcacgtccctacagtctgaagattttgcaacttattactgtcaacagtattacagtcacccgctcactttcggcggagggaccaaggtggagatcaaac |
| **P5B3** | Heavy chain | gaggtgcagctggtggagtccgggggaggcatagttcagcctggggggtccctgagactctcctgtgcagcctctggattcaccttcagtagctactggatgcactgggtccgccaagctccagggaaggggctggtgtgggtctcacgtataaatagtgatgggagtattataaactacgcggactccgtgaagggccgattcaccatctccagagacaacgccaagaagacgctgcatctgcaaatgaacagtctgagagccgaggacacggctgtatattactgtgcaaaagacggggtctacgatgccttagatatctggggccaagggacaatggtcaccgtctcttcag |
|  | Lambda light chain | tcctatgagctgactcagccaccctcggtgtcagtgtccccaggacagacggccaggatcacctgctctgcagatgcattgccaaaccaatatacttattggtatcagcagaagccaggccaggcccctgtgttgataatgtataaagacagtgagaggccctcagggatccctgagcgattctctggctccagctcagggacaacagtcacgttgaccatcagtggagtccaggcagaagacgaggctgactattactgtcaatcaacagacagccgtggtacctatgtggtattcggcggagggaccaagctgaccgtcctagg |
| **P5E10** | Heavy chain | gaggtgcagctggtggagtctgggggaggcctggtcaagcctggggggtccctgagactcacctgtgcagcctctggattcagtatcacgacctttaccatgggctgggtccgccagtctccagggaaggggctggagtgggtcgcatccattagtcccaccgcagacactttctacggagactcagtgaagggccgattcatcatctccagagacaacaccaagaactcactgtttctgcaaatggacagcctgagagtcgaggacacgtctgtatattactgtgcgagagatttagtttctttctggataaacttgtgggcccctacggacgcctggggccaagggaccacggtcaccgtctcctcag |
|  | Lambda light chain | gacatcgtgatgacccagtctccaggctccctggctgtgtctctgggcgagagggccaccatttcctgcaagtccagccagagtgttttttacagctccaacaatcagaactacttagcttggtaccagcagagaccaggacagcctcccaaactgctcatttactgggcatctacccgggagtccggggtccctgagcgattcagtggcagcgggtctgggacagatttcactctcaccatcagcagcctgcaggctgaagatgtggctgtttattactgtcaacaatattattcttctccgctcactttcggcggagggaccaaggtggagatcaaac |
|  |  |  |
|  |  |  |
|  |  |  |
| **Anti-PscF mAbs** |  | **Variable domain nucleotide sequence** |
| **P1D5** | Heavy chain | caggtgcagctgcatgaatcgggcccgggactggtggagccttcacagaccctgaccctcacctgcactgtctctggtggctccaccgacagtgatggcgtctactggacctggatccggcagcccgccgggaagggattggagtggattgggcgaatagataataggggcacatatagttataatccctccctcaggagtcgaatcaccatttcagaagaaccgtcccagaaccagttctccctgagggtgaggtctttgaccgccgcagatgcgggcgtctattattgtgcgagagaggtccgacgtctacaaggacggcgctactattattatggcaccgacgtctggggcccggggaccacggtcaccgtctcctcag |
|  | Kappa light chain | gatattgtgatgactcagtctccactttccttgtccgtcacccttggagagtcggcctccatctcctgcaggtctagccagagcctcctccatactaatgcatacaactatttggattggtacctgcagaggccagggcagtctccacagctcctgatctatttgagttctgttcgggcctccggggtccctgacaggttcagtggcaggggatcaggcacagattttacactgaaaatcagcagagtggaggctgaggatgttgggatttatttctgcatgcaagctctagaaatgccattcactttcggccctgggaccaaagtggatatcaaac |
| **P5G10** | Heavy chain | aggtgcagctacagcagtggggcgcaggactgttgaagccctcggagaccctgtccctcacctgcgctgtctatggcgggtcattcagtgactactactggacctggatccgccaggccccaggaaaggggctggagtggattggggaaaatagacacaggggtaccaccaactacaacccgtccctgaagggtcgagtcaccatatcagtagacacgtccaagaaccagttcaccctgaaggtgacctctgtgacggccgcggacacggctgtctacttctgtgcgaggcagaggggaagtagtggtggagcaccctccggtctggacgtctggggccaagggaccacggtcaccgtctcctcag |
|  | Lambda light chain | cagtctgtgctgactcagccaccctcagcgtctgggacccccgggcagagggtcaccatctcttgttctggagcctcgtccaacatcggaactaattatgtcttctggtaccagcagttcccaggaacggcccccaaactcctcatctataggaatggtcaccggccctcaggggtccctgaccgattctctggctccaagtctggcgcctcagcctccctggccatcagtgggctccggtccgacgatgaggctgattattactgtgcaacatgggatgacagcctgagttctcaggtcttcggcggagggaccaagctgaccgtcctaa |
| **P1B7** | Heavy chain | gaggtgcagctgttggagtctgggggaggcttggtgcagcctggggggtccctgagactctcctgtgcagcctctggattcacctttaaaagctatgccatgagctgggtccgccaggctccagggaaggggctggagtgggtctcagctattagtggcagtggtggtagcaaatactacgcagactccgtgaagggccggttcaccatctccagagacgattccaagaacacgctgtatctgcaaatgaacagcctgagagtcgaggacacggccgtttattactgtgcgaaacccttattagatagcgtgtactgcttggagtcctggggccagggaaccctggtcaccgtctcctcag |
|  | Kappa light chain | gacatccagatgacccagtctccatcctcactgtctgcatctgtaggagacagagtcaccatcacttgtcgggcgagtcaggacattagcaattatttatcctggtatcagcagagaccagggaaagcccctaagtccctgatctatgctgcattcagattgcaaagtggggtcccatcaaagttcagcggcagtggatctgggacagatttcactctcaccatcagcagcctgcagcctgaagattttgcaacttattactgccaacagtattatacttaccctcctacttttggccaggggaccaagctggagatcaaac |
| **P5D5** | Heavy chain | gaggtgcagctgttggagtctgggggaggcttggtacagcctggggggtccctgagactctcctgtgcagcctctggattcacctttaacaactatgccatgagctgggtccgccaggctccagggaaggggctggagtgggtctcagcaattagtggtagtggtggaagcaaatactacgcagactccgtgaagggccggttcaccatctccagagacgattccaagagcaggctgtatctgcaaatgaacagcctgagagtcgaggacacggccttatattactgtacgaaacccttattagaaagcgtgtactacttggagtcctggggccagggaaccctggtcaccgtctcctcag |
|  | Lambda light chain | gacatccagatgacccagtctccatcctcactgtctgcatctgtaggagacagagtcaccatcacttgtcgggcgagtcagggcattagcaatcatttagcctggtttcagcagaaaccagggaaagcccctaagtccctgatctatgctgcatcccgtttgcaaagtggggtcccatcaaggttcagcggcagtggatctgggacagatttcactctcaccatcagtagcctgcagcctgaagattttgcaagttattactgccaacagtattatagttaccctccagcttttggccaggggaccaagctggagatcaaac |
| **P1B4** | Heavy chain | gaggtgcagctgttggagtctgggggaggcttggtgcagcctggggggtccctgagactctcctgtgcagcctctggattcacgtttagcagttatgccatgaactgggtccgccaggctccagggaaggggctggagtgggtctcagttattagtggcagcggtggtagcacatactacgcagactccgtgaagggccggttcaccatctccagagagaactccaagaacacgctgtatctgcaagtgaacagcctacgagccgaggacacggccgtttactaccgtgcgaaagatctacggtgtagtggcactggctgcccctacggtatggacgtctggggccaagggaccacggtcaccgtctcctcag |
|  | Kappa light chain | gaaattgtgttgacgcagtctccaggcaccctgtctttgtctccaggggaaaaagccaccctctcctgcagggccagtcagagtgtttccagcaactacttagcctggtaccagcagaaacctggccaggctccccggctcctcgtctatggttcatccagcagggccacaggcatcccagacaggttcagtggcagtgggtctggggcagacttcactcttcaaatcagcaaactggagcctgaagattttgcagtgtattactgtcagcagtacggtagctcgcccttattcactttcggccctgggaccaaagtggatatcaaac |
| **P1F5** | Heavy chain | gaaattgtgttgacgcagtctccaggcaccctgtctttgtcttcaggggaaagagccaccgtctcctgcagggccagtcagagtgttagcgccagcaatttagcctggtaccagcagaaacctggccaggctcccaggctcctcatatatggagtatccagcaggcccactggcatcccagacaggtttagtggcagtgggtctgggacagacttcactctcaccgtcagcagactggagcctgaagattttgcggtgtattactgtcagcagtatggtagttcaccgacgttcggccaagggaccaaggtggagatcaaac |
|  | Lambda light chain | gatattgtgatgactcagtctccactctccctgcccgtcacccctggagagccggccgccatctcctgcaggtctagtgagagtctcctctatagtaatggagacaactatttggattggtatgtgcagaagccagggcagtctccacaactcctgatctatttgggttctaagcgggcctccggggtccctgacaggttcagtggcagtggatcgggcacacattttgtactaaaaatcagcagagtggaggctgatgatgttggtgtttattactgcatgcaagctctacaaagtcctccgtacacttttggcccggggaccaagctggagatcaaac |
| **P1D7** | Heavy chain | gaggtgcagctgttggagtctgggggaggcttggtacagcctggggggtccctgagactctcctgtgcagcctctggattcacctttaccagctatgccatgagctgggtccgccaggctccagggaaggggctggagtgggtctcagctctcagtaatagtggtgatagcacatactacgcagactccgtgaagggccggttcaccatctccagagacaattccaagaacacgctgtatctgcagatgcacagcctgagagtcgaggacacggccgtatattactgtgcgaaagatcgggaaggtgactacggtgactacgtatttgactactggggccagggaaccctggtcaccgtctcctcag |
|  | Kappa light chain | tcctatgtgctgactcagccaccctcggtgtcagtggccccaggacagacggccaggattccctgtgggggaaacaatattggaaggaaaagtgtgcactggtaccagcagaagccaggccaggcccctgtgctggtcgtctatgatgatagcgatcggccctcagggatccctgagcgattctctggctccaactctgggaacacggccaccctgaccatcagcagggtcgaagccggggatgaggccgactattactgtcaggtgtgggatagtagtgatgatcttgtggtattcggcggagggaccacgctgaccgtcctag |
| **P3G2** | Heavy chain | caggtgcagctgcaggagtcgggcccaggactggtgaagccttcagagaccctgtccctcacctgcagtgtctctggtggctccgtcgcaagtggcgatttctactggagctgggtccggcagcccgccgggacgggactggagtggattgggcgtgtctcgaccagtgggagcaccaactgcaacccctccctcaagagtcgagtcaccatatcaattgacacgtccaagaacgagttctccttgaggctgccctctgtgaccgccgcagacacggccgtttactactgtgcgagatatcgccccgggtccgggggcctaggagtctactatgactactggggccagggaatcctggtcaccgtctcctcag |
|  | Lambda light chain | cagtctgccctgactcagcctccctccgcgtccgggtctcctggacagtcagtcaccatctcctgcactggagccagcagtgacattggtggttataactatgtttcctggtaccaacaacacccaggcagagcccccagactcatgatttatgaggtccataaccggccctcaggggtccctgatcgtttctctggttccaagtctggcaacacggcctccctgaccgtctctgggctccaggctgaggatgaggctgattattactgcatgtcgtatgcaggcggcaacattttgcttttcggtggagggaccaagttgaccgtcctag |
| **P3G6** | Heavy chain | gaggtgcagctgttggagtctgggggaggcttggtacagcctggggggtccctgagactctcctgtgcagcctctggattcacctttagcagctatgccatgagctgggtccgccaggctccagggaaggggctggagtgggtctcagctattagtggcagcggtggtagcacatactacgcagactccgtgaagggccggttcaccatctccagagacaattccaagaacacgctgtatctgcaaatggacagcctgagggccgaggacacggccgtatattactgtgcgaaagatctacgctgtagtggtactggctgcccctacggtatggacgtctggggccaagggaccacggtcaccgtctcctcag |
|  | Kappa light chain | gaaattgtgttgacgcagtctccaggcaccctgtctttgtctccaggggaaagagccaccctctcctgcagggccagtcagagtgttagcagcagctacttagcctggtaccagcagaaacctggccaggctcccaggctcctcatctatggtgcatccagcagggccactggcatcccagacaggttcagtggcagtgggtctgggacagacttcactctcaccatcagcagactggagcctgaagattttgcagtgtattactgtcagcattatggtagctcactttacacttttgcccaggggaccaaggtggaaatcaaac |
| **P3G7** | Heavy chain | gaggtgcagctggtggagtctgggggaggcttggtccagcctggacggtccctgacactctcctgtgcagtcactggattcatcgtcagagactaccacatcgactgggtccgccaggctccagggaagggactggagtgggttggccgtgcgagaaataaagctagccgttatagggtagtttacgccgcgtctctgaaggacagattcaccattattagagatgactcaaagaactcaatatacctgcgaatgagcagcctgaaaaccgaggacgcggccatgtatttttgtgttagagttggtcactatgatacgcaaggttatgccctggatgattatgatgtctggggccaagggacagcggtcaccgtctcctcag |
|  | Lambda light chain | cagtctgtgttgacgcagtcgccctcagtttctgcggccccaggacagagggtcaccatccactgttttggaagcagctccaccattggaagtaatcaattatcctggtaccagcaagtcccagggacagcccccaaacttctcatttatgacagtgacaagcgaccctcagggattcctgaccgattctccgcctccaagtctggcacatcaggcaccctggatatcagcggcctccagagtggggacgaggccgattattactgcgagacatgggatgacaccctgaaagctgtcgttttcggcggggggaccaagctgaccgtcctag |
